# Accurate Prediction of the Response of Freshwater Fish to a Mixture of Estrogenic Chemicals

**DOI:** 10.1289/ehp.7598

**Published:** 2005-03-14

**Authors:** Jayne V. Brian, Catherine A. Harris, Martin Scholze, Thomas Backhaus, Petra Booy, Marja Lamoree, Giulio Pojana, Niels Jonkers, Tamsin Runnalls, Angela Bonfà, Antonio Marcomini, John P. Sumpter

**Affiliations:** ^1^Institute for the Environment, Brunel University, Uxbridge, Middlesex, United Kingdom; ^2^Centre for Toxicology, School of Pharmacy, London, United Kingdom; ^3^Department of Biology and Chemistry, University of Bremen, Bremen, Germany; ^4^Institute for Environmental Studies, Vrije Universiteit, The Netherlands; ^5^Department of Environmental Sciences, University of Venice, Venice, Italy

**Keywords:** concentration addition, estrogen, estrogen mimic, fathead minnow, mixture effects, *Pimephales promelas*, prediction

## Abstract

Existing environmental risk assessment procedures are limited in their ability to evaluate the combined effects of chemical mixtures. We investigated the implications of this by analyzing the combined effects of a multicomponent mixture of five estrogenic chemicals using vitellogenin induction in male fathead minnows as an end point. The mixture consisted of estradiol, ethynylestradiol, nonylphenol, octylphenol, and bisphenol A. We determined concentration–response curves for each of the chemicals individually. The chemicals were then combined at equipotent concentrations and the mixture tested using fixed-ratio design. The effects of the mixture were compared with those predicted by the model of concentration addition using biomathematical methods, which revealed that there was no deviation between the observed and predicted effects of the mixture. These findings demonstrate that estrogenic chemicals have the capacity to act together in an additive manner and that their combined effects can be accurately predicted by concentration addition. We also explored the potential for mixture effects at low concentrations by exposing the fish to each chemical at one-fifth of its median effective concentration (EC_50_). Individually, the chemicals did not induce a significant response, although their combined effects were consistent with the predictions of concentration addition. This demonstrates the potential for estrogenic chemicals to act additively at environmentally relevant concentrations. These findings highlight the potential for existing environmental risk assessment procedures to underestimate the hazard posed by mixtures of chemicals that act via a similar mode of action, thereby leading to erroneous conclusions of absence of risk.

Many environmental contaminants are capable of disrupting endocrine function in humans and wildlife. This phenomenon has been associated with reduced fecundity, reproductive failure, and population-level effects in a variety of aquatic organisms (Jobling et al. 2002; Matthiessen and Gibbs 1998; Nash et al. 2004). This highlights the urgent need to develop accurate methods of assessing the risk that these chemicals pose. Current methods usually focus on the assessment of single chemicals. This is in clear contrast to real-world exposure situations, which are generally to mixtures of endocrine-disrupting chemicals, many of which act via a common mode of action. This means that the overall risk posed in real exposure situations may be greater than that expected on the basis of the effects assessment of the individual mixture components, due to the potential for combined effects. Concerns over the ecological significance of these effects were heightened in the late 1990s after reports of spectacular synergisms between binary mixtures of estrogenic pesticides *in vitro* (Arnold et al. 1996). These results were subsequently withdrawn because of issues of reproducibility, leading many to question the overall significance of mixtures (Kortenkamp and Alterburger 1999). However, the issue has continued to attract interest in view of the fact that many of the estrogenic effects reported in the literature exceed expectations based on chemical-by-chemical assessments. A notable example of this is the discrepancy between the widespread distribution of reproductive abnormalities in wild fish populations relative to the low concentrations of estrogenic chemicals to which they are exposed (Jobling et al. 1998; van Aerle et al. 2001).

Many of the chemicals identified as endocrine disruptors are known to mediate their effects by binding with the estrogen receptor (Payne et al. 2000). Estrogenic chemicals include both the natural and synthetic steroidal estrogens, as well as a wide range of synthetic chemicals that mimic the actions of endogenous estrogen. The potencies of these different types of chemical vary over several orders of magnitude. For example, the steroidal estrogens, such as 17β-estradiol (E_2_) and 17α-ethynylestradiol (EE_2_), are capable of exerting estrogenic effects on fish when present in the water in the low nanograms per liter range (Thorpe et al. 2003). These chemicals pose a significant environmental risk, having been detected in effluents that discharge into rivers at concentrations that are individually capable of inducing a significant effect (Desbrow et al. 1998). In contrast, chemicals that mimic the actions of estrogen, such as the alkylphenols, exhibit much lower potencies and rarely occur at concentrations that are individually effective in the environment (Desbrow et al. 1998). Hence, the individual assessment of the hazard posed by these chemicals indicates a negligible risk. However, this approach does not account for the potential for endocrine disruptors to act in combination. This may lead to the underestimation of hazards that exist in real exposure situations, resulting in erroneous conclusions of absence of risk.

Increasing recognition of these shortcomings has prompted considerable efforts to investigate the combined effects of estrogenic chemicals (e.g., Ashby et al. 1997; Soto et al. 1994). However, many of these studies have been hampered by inadequate theoretical foundations on which to base the expected effects of mixtures of chemicals that exhibit nonlinear concentration–response curves (Kortenkamp and Altenburger 1999). More recently, however, the pharmacological concept of concentration addition (CA) has been applied to the assessment of estrogenic mixtures. This concept is based on the assumption that the components of the mixture act in a similar manner, such that replacing one or more chemicals totally, or in part, with the other mixture components can produce the same overall effect. The overall effect of the mixture can therefore be described quantitatively using a mathematical model, based on the concentration and potency of the individual mixture components (Bödeker et al. 1992). This means that potential hazards can be predicted from basic information about the components of the mixture and its composition (number and concentration of chemicals present), thereby negating the need for mixture testing. A number of studies have attempted to validate this concept by comparing the effects of the mixture with those expected on the basis of additivity. This has involved the single-substance testing of the individual mixture components in order to gain information for the modeling of mixture effects. The predictions made can then be tested experimentally. This approach has been used extensively in aquatic toxicology to demonstrate the validity of CA as a means of predicting the toxicity of multicomponent mixtures of similarly acting compounds in various assays with fish, daphnia, algae, and bacteria (e.g., Altenburger et al. 2000; Backhaus et al. 2000, 2004; Faust et al. 2001; Hermens et al. 1984a, 1984b; Könemann 1981).

There is considerable evidence that CA may also be used to predict the effects of mixtures of estrogenic chemicals. The validity of this approach has been demonstrated *in vitro*, using assays such as the yeast estrogenicity screen (YES) and the human breast cancer cell proliferation assay (E-SCREEN) (Payne et al. 2000, 2001; Rajapakse et al. 2002; Silva et al. 2002). Such studies have revealed the capacity for the components of the mixture to contribute to the overall effect by acting in relation to their potency, even at low-effect concentrations. For example, Silva et al. (2002) combined eight estrogenic chemicals at low-effect concentrations and demonstrated that the effects of this mixture were consistent with the predictions of CA. This highlights the capacity for these chemicals to act in combination, even when the individual components of the mixture are present at concentrations below the threshold of statistically detectable effects. This has become known as the “something from nothing” phenomenon (Silva et al. 2002).

In light of this *in vitro* evidence, there is now an urgent need to assess whether these mixture effects also occur in higher life forms, which reflect the net effects of complex chains of events involving the uptake, distribution, and metabolism of test agents until they reach their target sites. The induction of the egg yolk protein vitellogenin (VTG) is an established *in vivo* assay for analyzing estrogenic effects in fish. This protein is normally induced in the livers of female fish in response to stimulation by endogenous estrogen. However, it can be induced in both male and female fish exposed to extremely low concentrations of estrogenic chemicals (Sumpter and Jobling 1995). Although a causal relationship has not been established, a number of studies have demonstrated that VTG induction is associated with effects at higher levels of biological organization (e.g., Harries et al. 2000). It therefore offers a sensitive and integrated measure of estrogenic activity, which is relevant to the assessment of environmental risk. Recent evidence indicates that the induction of VTG can be used to assess the joint action of binary mixtures of estrogenic chemicals *in vivo* (Thorpe et al. 2001, 2003). Here, we have applied this assay to the analysis of multi-component mixture effects.

The aim of this study was to investigate the predictability of the combined effects of five estrogenic chemicals on VTG induction in the fathead minnow (*Pimephales promelas*). We used CA as a concept on which to base the expectation of additivity. We tested the predictive power of CA by analyzing the estrogenic effect of each mixture component individually. Information on their potency was then used to make predictions, which were then tested by comparison with the observed mixture effects. Mixture effects at low-effect concentrations of the individual components were also investigated to analyze the applicability of CA under environmentally realistic conditions. All studies were conducted using an optimal experimental design that minimized the number of test organisms. Quality checks of the exposure conditions were conducted using analytical chemistry.

The work described in this article contributes to our current understanding of the combined effects of multicomponent mixtures of estrogenic chemicals at higher levels of biological complexity, as well as aiding in the development of methods that can be applied to the analysis of mixtures. Hence, the findings are of considerable relevance to the assessment of environmental risk.

## Materials and Methods

### Test organisms.

A stock of fathead minnows was obtained from Osage Catfisheries (Osage Beach, MO, USA). These fish, and their offspring, were used to conduct 14 independent exposure studies. Before exposure, stock fish were held in communal holding tanks with a recirculating water supply. The exposure studies were conducted in 30-L glass aquaria (0.6 m × 0.3 m × 0.3 m), which were supplied with a continuous flow of water. The analysis of VTG induction focused on the responses of male fish. However, an equal number of females were included in each experiment to reduce the level of aggression between males. During the exposure, the fish were fed twice daily: once with frozen brine shrimp and once with flaked fish food. The photoperiod was maintained at 16-hr light/8-hr dark with 20-min dawn and dusk transition periods.

### Test chemicals.

We investigated the activity of five estrogenic chemicals. These were selected on the basis of previous reports of their presence in the environment and because of their likely association with intersexuality in wild fish (Desbrow et al. 1998). They included the natural steroidal estrogen E_2_, the synthetic steroidal estrogen EE_2_, and the estrogen-mimicking compounds 4-*tert*-nonylphenol (NP), 4-*tert*-octylphenol (OP), and bisphenol A (BPA). Stocks of E_2_ (98% purity), EE_2_ (98% purity), OP (97% purity), and BPA (99% purity) were purchased from Sigma Aldrich (Dorset, UK). NP (99% purity) was obtained from ACROS Organics (Leicestershire, UK). All chemicals were dissolved in HPLC-grade dimethylformamide (DMF) supplied by BDH Laboratory Supplies (Dorset, UK).

### Water supply and test apparatus.

We applied stock solutions to the tanks using a Watson-Marlow 205U multichannel peristaltic pump using silicon tubing (Watson Marlow, Falmouth, Cornwall, UK). Solutions were delivered at a rate of 0.02 mL/min into mixing vessels, which were supplied with dechlorinated water that had been heated to 25°C. Water entered the mixing vessels at a flow rate of 300 mL/min, resulting in a 1:15,000 dilution of the stock solution. The diluted stock solution then flowed into the tanks at a rate of 18 L/hr, which resulted in one complete water change every 100 min. Dissolved oxygen and water temperature were recorded daily, and the functioning of the delivery system was monitored throughout the study.

Delivery of the test chemical commenced 1 week before the start of each exposure. During this equilibration period, the fish were acclimatized to the experimental conditions in an identical set of undosed tanks. After 7 days, the fish were transferred into the tanks containing the chemical or chemicals, where they were maintained under exposure conditions for a period of 2 weeks. Three control tanks were run alongside each exposure. Two of these were negative controls (NCs), consisting of one undosed tank that received water only [water control (WC)] and one tank that was dosed with DMF [solvent control (SC)]. A positive control (PC) was also included in each study. The PC tank was dosed with EE_2_ at a concentration of 10 ng/L, which has previously been found to induce a maximum VTG response (Panter et al. 2002).

### Analytical chemistry.

We determined exposure concentrations at three different time points during each experiment. We collected the first set of water samples after 1 week of dosing, immediately before the addition of the fish. The second set was taken 1 week after this, and the third set was taken after the final week, on the day that the exposure was terminated. Water samples were collected in solvent rinsed glass bottles. If the sample was to be analyzed for the presence of steroid estrogens, the bottles were silylated before use. The water samples were then analyzed according to the nature of the chemical in question, using one of the four following analytical techniques.

Water samples containing EE_2_ were extracted onto preconditioned solid-phase C18 cartridges. Extracts were eluted into methanol, which was removed under a stream of nitrogen. The extracts were then resuspended in ethanol, and the EE_2_ concentration was determined using an established radioimmunoassay technique (Lange et al. 2001). Samples containing E_2_ also underwent solid-phase extraction (SPE) on a DVB Speedisk (Baker, Deventer, The Netherlands). After cleanup of the extracts with C18 cartridges, derivitization of E_2_ was carried out using silyl reagents before analysis using gas chromatography combined with ion trap detection (adapted from Belfroid et al. 1999; Houtman et al. 2004). Samples containing BPA underwent SPE analogous to the E_2_ procedure, after which the extracts were analyzed using HPLC coupled to diode array detection. This was carried out under isocratic elution conditions with methanol/water (60/40, vol/vol) (adapted from Belfroid et al. 1999). For the analysis of water samples containing NP and OP, the extraction step was omitted. After the addition of acetonitrile (5%), large sample volumes (300–800 μL) were injected onto a reversed-phase HPLC column, which was coupled with an ion trap mass spectrometer via an electrospray interface for on-column enrichment. Analytes were eluted using a fast gradient (Pojana et al. 2004).

### Experimental design.

We determined the complete concentration–response curve of each chemical in the test system in order to provide the information necessary to generate the prediction. The successful comparison of observed and predicted mixture effects was dependent upon the quality of these data. In order to generate a prediction of low uncertainty, that is, high accuracy and precision, it was necessary to minimize the chance of unknown systematic shifts in VTG sensitivity for each chemical within the study time that could result in a biased prediction (inaccuracy), and determine the concentration-effect information of each compound with a certain precision in order to maintain a given statistical variability of the prediction (precision). We achieved this by repeating each exposure at least once after a given time lag. Data from repeated studies were then pooled.

Slight differences in the absolute VTG levels between studies were accounted for by standardizing the absolute effects scale to relative effects of between 0 and 1. The mean VTG concentration in the fish from the NC (SC) and the PC tanks were used as the minimum and maximum responses, respectively. This scaling was carried out after the VTG effects data were log_10_ transformed, such that a median effective concentration (EC_50_) corresponds to the concentration that produces a log_10_-transformed VTG induction, which is median in relation to the NCs and PC.

The aim of the single-chemical exposures was to produce the data necessary to predict the median effect concentration of the mixture without exceeding a given level of statistical uncertainty (the 95% confidence limits for the predicted EC_50_ were set at a maximum of ± 0.2 on the log_10_-transformed concentration scale). This relied on the premise that there was average effect data variability, determined on the basis of historical data sets produced under similar test conditions, and it required that the concentration range tested provided sufficient information on the VTG response curve. This information was based on results of the repeated preliminary exposure studies, each of which included six different concentrations, to which four male and four female fish were exposed.

In order to compare the mixture effects with the predictions of CA for a wide range of different VTG levels in the final mixture experiment, we used a “fixed-ratio” mixture design: a master stock was prepared, containing each of the chemicals at their EC_50_ concentrations. This was diluted to give a range of mixture concentrations of 100, 50, 30, 20, 10, and 5%, which corresponded with relative VTG responses between 0 and 100%, according to the CA expectations. Fish were exposed to this dilution series in two independent studies using the same methods and design as employed in the individual exposure studies. The concentration–response to the mixture was then determined and related to the effects predicted by CA.

In order to directly relate the effects of the compounds to the observed mixture effects, we performed a second mixture experiment. The design of this experiment involved the parallel testing of each chemical, both individually and in combination. Only one concentration of each chemical was tested. This approach aimed to investigate the potential for mixture effects to occur at low-effect concentrations of the components, that is, at concentrations that would not, individually, induce a statistical significant effect (Silva et al. 2002). The low-effect concentrations adopted were based on the EC_50_ of each chemical divided by 5. According to the principles of CA, it was predicted that this mixture would induce a 50% level of effect.

### Fish sampling and analysis of plasma VTG.

At the end of each exposure, the fish were sacrificed by overdosing with MS222 (Sigma Aldrich). The length and weight of each individual were recorded before bleeding. Blood samples were collected from the caudal peduncle using heparinized capillary tubes (Hawksley and Sons Ltd., Sussex, UK). These were centrifuged at 4,000*g* for 5 min. Plasma was then drawn off and stored at −20°C until required for analysis. Plasma VTG concentrations were determined using a carp-VTG enzyme-linked immunosorbent assay (ELISA) validated for the measurement of VTG in fathead minnows (Tyler et al. 1996).

### Mathematical modeling and statistical analysis.

We determined concentration–response curves for each of the five chemicals and for the mixture using pooled data from the repeated exposures. To account for the intra- and interexperimental variability associated with this nested data scenario, we used the generalized nonlinear mixed modeling approach in which both fixed and random effects are permitted to have a nonlinear relationship with the effect end point (Vonesh and Chinchilli 1997). As random effect, a shift parameter was included in the nonlinear regression model, which accounts for a shift of the whole curve based on the log_10_-transformed concentration scale. Furthermore, a best-fit approach was adopted: three different regression models (probit, logit, and Weibull) were fitted independently to the same pooled data set, and the best fit was selected on the basis of statistical criteria (Scholze et al. 2001). This approach was implemented using the NLMIXED function of the SAS statistical software package (SAS Institute, Cary, USA).

The expected concentration–response relationship of the mixture was calculated using CA, which is represented by Equation 1:


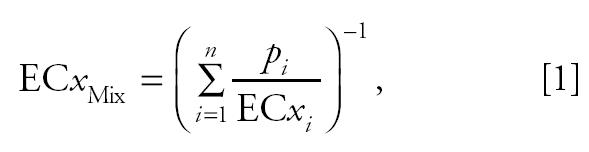


where EC*x*_Mix_ is the concentration of the mixture that induces an overall effect *x*, EC*x_i_* is the concentration of the *i*th chemical in an *n*-component mixture required to induce the same magnitude of effect, and *p_i_* is the proportion of the *i*th component in the mixture (Backhaus et al. 2000). Hence, in addition to information regarding the exact composition of the mixture, knowledge of identical effect concentrations (EC*x* values) of the mixture components is all that is required to predict an EC*x* value for the mixture. CA was used to predict EC*x* values for the mixture in steps of 1% for effect levels from 10% up to 95%. These values were then connected using straight lines to give a graphical representation of the predicted curve. All predicted effect concentrations are estimates and are therefore subject to stochastic variability, which meant that the predicted effect concentration of the mixture also had to include a measure of statistical uncertainty. This was achieved using the bootstrap method (Efron and Tibshirani 1993), which enabled 95% confidence limits to be derived for the mean predicted effect.

## Results

### Analytical determination of exposure concentrations.

Because of occasional technical problems that were encountered with the analysis of the water samples, we did not obtain full sets of reliable data for each chemical in all exposures. Inconsistencies between data sets for some chemicals created problems when plotting the concentration–response data by effectively shifting the position of the curve along the x-axis, thereby increasing the variability associated with the biological response. This reduced the accuracy and precision of the effect model for the chemicals concerned. In contrast, when the biological data (the VTG response) were plotted against the nominal concentrations, it proved to be highly reproducible. This strongly suggested that the occasional differences between nominal and measured concentrations were artifactual. For this reason, the concentration–response analyses were based on nominal, as opposed to measured, exposure concentrations.

The problems encountered with the chemical analyses were subsequently resolved, and good agreement between the nominal and actual exposure concentrations of each chemical was obtained in the mixture experiments. This is demonstrated in Table 1, which shows the measured concentration of all of the mixture components on the first day of each of the mixture experiments. These values were between 100 and 166% and 66 and 128% of the nominal value for EE_2_ and E_2_, and 64 and 128%, 50 and 110%, and 55 and 105% of nominal value for NP, OP, and BPA, respectively. Hence, the extent of the deviation from nominal concentrations did not vary consistently between chemicals, despite the differences between their exposure concentrations. The mean measured concentration of each chemical remained fairly constant over time: the measured concentrations of EE_2_, E_2_, NP, OP, and BPA 1 week and 2 weeks after the start of the exposure were an average of 99 and 77%, 89 and 92%, 92 and 96%, 84 and 97%, and 92 and 86% of those measured at the start of the exposure, respectively. Hence, the analytical data generally confirm that the exposure conditions were similar and reproducible for each of the chemicals used.

### Biological effects data.

All exposure studies ran to completion. The rate of mortality did not differ between treatments, which indicated that the chemicals tested were not acutely toxic and that the fish were not unduly stressed. The baseline concentrations of VTG determined for control males and females were consistent with the literature (Harries et al. 2000; Panter et al. 1998; Tyler et al. 1996), and there were no significant differences between the VTG levels of WC and SC fish in any of the exposures. Clear concentration–response curves could be determined for male fish in response to each of the single chemicals as well as to the mixture. In contrast, female VTG levels exhibited extensive variability, depending on their stage in the spawning cycle (data not shown). For this reason, only the data from male fish were suitable for inclusion in the analyses.

### Concentration–response analysis for individual chemicals.

The analysis of the concentration–response data for each chemical was based on data pooled from at least two independent exposure studies. In the case of OP and BPA, a third smaller-scale study was conducted. This was necessary because the first two exposures did not cover the full extent of the VTG response curve. In general, data from repeated studies showed excellent agreement, although there was some disparity between the positions of the curves for EE_2_ and, to a lesser extent, E_2_. This is likely to reflect the increased potential for error when working in the nanograms per liter concentration range. These findings support the need to base the prediction of mixture effects on more than one set of data using the means of repeated and pooled data sets.

**Figure 1:**
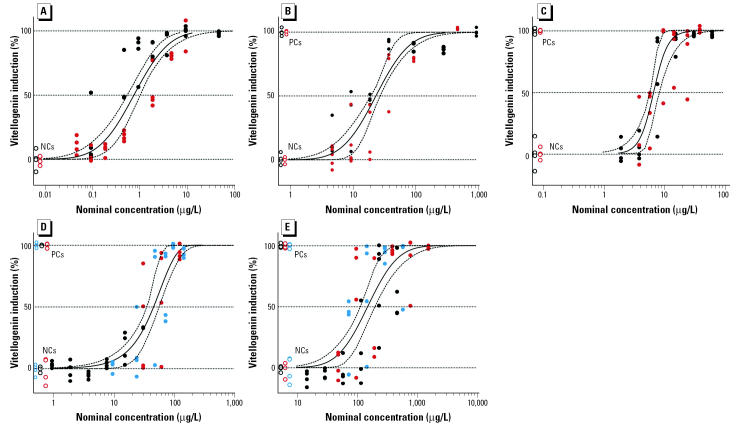
Pooled concentration–response data and best-fit regression curves for each of the individual mixture components. (*A*) EE_2_. (*B*) E_2_. (*C*) NP. (*D*) OP. (*E*) BPA. Each point represents the VTG response of one fish, with each color representing an independent exposure study. The solid line represents the best-fit curve, and the dashed lines represent the 95% confidence interval.

**Figure 2:**
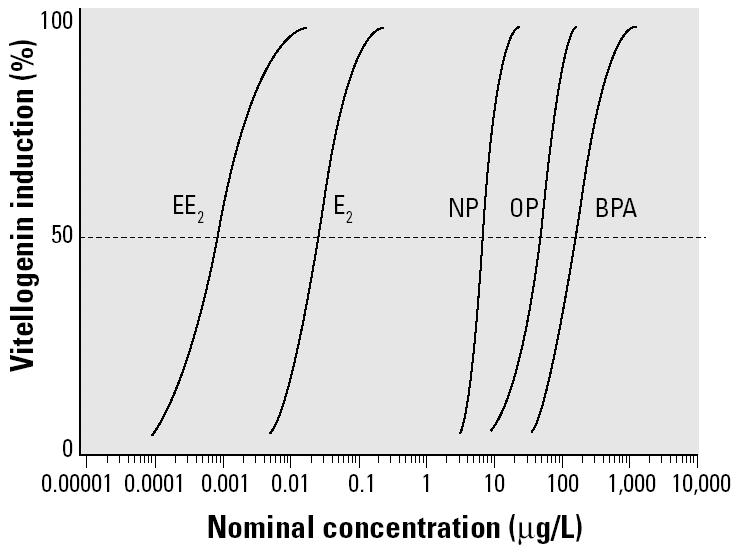
Best-fit regression curves for the individual mixture components plotted on the same concentration scale.

Each of the chemicals tested induced VTG in a concentration-dependent manner. Figure 1 shows the concentration–response data for each chemical and their estimated regression curves. The corresponding best-fit models with estimated parameters are given in Table 2, together with the estimated EC_50_ values and the confidence limits, which were always below the planned tolerance benchmark of ± 0.2 on the log_10_-transformed concentration scale. It was possible to determine the 100% effect (relative to the PC) for each chemical, and the lowest tested concentration did not provoke effects significantly different from the untreated controls. This allowed the estimation of full concentration–response curves without needing to extrapolate to untested effect levels. Figure 2 shows the concentration–response curves for each chemical plotted on the same concentration scale, thus highlighting the magnitude of variations in potency. EE_2_ was the most potent chemical tested, with an EC_50_ of 0.9 ng/L, which was between 25 and 30 times more potent than E_2_. The EC_50_ of the natural steroid E_2_ was 25 ng/L. NP and OP were 280 and 1,800 times less potent than E_2_, with EC_50_ values of 7 and 45 μg/L, respectively. BPA was the least potent chemical tested, with an EC_50_ of 150 μg/L. This was 6,000 times less potent than E_2_.

### Concentration–response analysis for the mixture.

**Figure 3:**
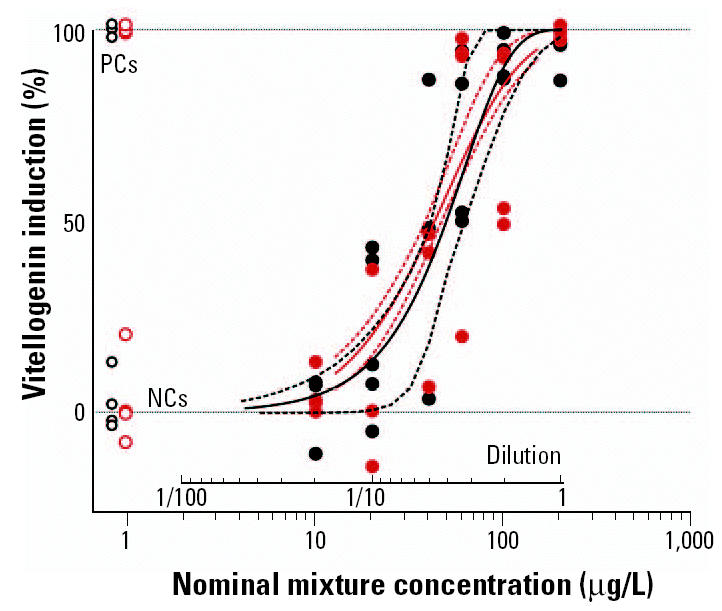
Comparison between the observed and CA-predicted mixture effects of five estrogenic chemicals in the male fathead minnow. Each point represents the VTG response of one fish, with each color representing an independent exposure study. The solid black line represents the best-fit of the observed effect data, and the solid red line represents the CA prediction. Dashed lines represent the 95% confidence intervals. The predicted effect of the mixture falls within the 95% confidence interval of the observed data across the entire dose–response curve.

The VTG response induced by the mixture is shown in Figure 3, together with the line of best fit and the curve predicted by CA. The variability associated with the best-fit estimate is shown in Table 2. A concentration–response curve was evident, and there was excellent agreement between the results of the two independent exposures. The pooled data sets provide sufficient information for EC estimates of low statistical uncertainty and thus a good basis for the comparative assessment of observed and predicted mixture effects. The comparison of the observed VTG response and the corresponding regression fit with the prediction curve yielded excellent agreement, independently of the effect level. No statistical deviation could be detected, with the prediction lying within the narrow 95% confidence limits along the full length of the curve. These findings provide evidence that estrogenic chemicals act in an additive manner *in vivo* and that their effects can be predicted accurately using CA.

### Mixture effects at low-effect concentrations.

**Figure 4:**
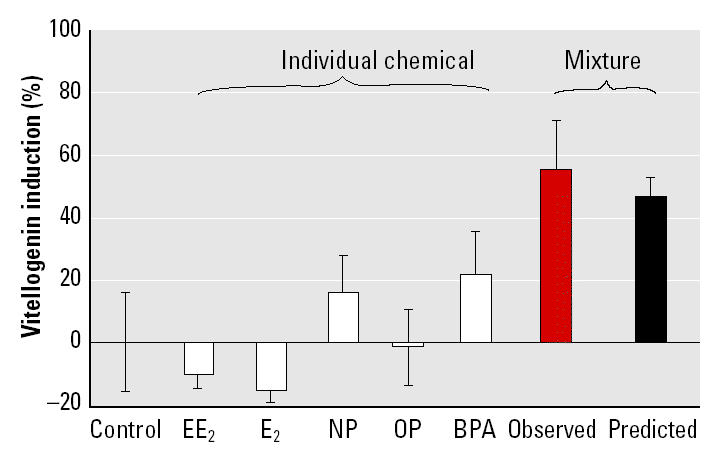
Mixture effects at low-effect concentrations (one-fifth of EC_50_) of five estrogenic chemicals. Error bars indicate SEM. Individual concentrations were 0.12 ng/L EE_2_, 5 ng/L E_2_, 1.4 μg/L NP, 9 μg/L OP, and 30 μg/L BPA. The mixture treatment contained all five chemicals at the aforementioned concentrations, resulting in an overall mixture concentration of 40.4 μg/L. Analysis of variance detected a significant difference between treatments (*F*_6,19_ = 4.05, *p* < 0.01). Post hoc tests revealed no difference between the response of fish exposed to each of the chemicals individually and that of the control fish. In contrast, the mixture elicited a response that was significantly different from that of the controls.

The results of the investigation into mixture effects for compounds at low-effect concentrations are shown in Figure 4. Analysis of the data revealed that, individually, each of the chemicals failed to provoke a response that was statistically different from that of the controls at a concentration that was equivalent to one-fifth of their EC_50_. In contrast, when fish were exposed to the same dose of all five chemicals in combination, VTG was significantly induced. In line with the first experiment, there was good agreement between the observed effect of the mixture and the prediction of CA, with the prediction falling within the confidence limits of the observed effects. This confirms that the combined action of estrogenic chemicals does not deviate from additivity even in the low-effect concentration range.

## Discussion

### Exposure concentrations.

The decision to determine the concentration–response relationships on the basis of nominal, as opposed to measured, exposure concentrations was made in order to overcome problems that were initially encountered with the analytical chemistry (discussed above). In theory, the measured concentrations should provide a more accurate reflection of the exposure conditions, because they account for experimental errors that may have arisen because of inaccuracies in the preparation of stock solutions and/or the dosing of tanks. As a result, the measured concentrations should provide the basis for the mathematical modeling of mixture effects. However, if problems occur when measuring the exposure concentrations, these can add more variability than they remove. This, in turn, reduces rather than improves the accuracy of the prediction. Hence, in the absence of a full set of reliable measured concentrations, it was more accurate to base the mathematical model on the nominal values.

This approach did not appear to reduce the reproducibility of the concentration–response analysis of NP, OP, and BPA. In contrast, the agreement between the concentration–response curves determined for E_2_ and EE_2_ in each of the repeated exposures was slightly reduced when the VTG response data were plotted against nominal, as opposed to measured, concentrations. However, these differences were marginal. This indicates that the nominal values provided a reliable indication of the real exposure concentrations and validates their use in the concentration–response analyses. This approach may not have used the chemical analytical data to their full potential. However, the determination of exposure concentrations was extremely useful in confirming the accuracy of the dosing system. Without this, it would not have been possible to validate the methods employed.

### Single-substance effects.

Despite the plethora of published data describing the potency of the chemicals tested in this study, comparisons between studies are complicated by apparent differences between the species tested, the end points analyzed, and the assay systems used. However, comparable studies involving the analysis of VTG induction in male fathead minnows exposed to estrogenic chemicals under flow-through conditions have yielded results that are consistent with the effects reported here. For example, Panter et al. (1998) reported the induction of VTG in response to between 32 and 100 ng/L of E_2_ after a 3-week exposure, which is in the same order of magnitude as the potency observed in this study. EE_2_ has previously been found to induce VTG at concentrations between 0.1 and 1 ng/L (Pawlowski et al. 2004). This is consistent with the EC_50_ of 0.9 ng/L reported here. The potency of NP is also consistent with previous evidence that this chemical is effective at concentrations between 1 and 10 μg/L in fathead minnows after a 2- to 3-week exposure (Harries et al. 2000; Pickford et al. 2003). Studies by Sohoni et al. (2001) suggest that BPA is less potent, although the effects reported were of a similar order of magnitude as those observed in this study. Concentration–response data from comparable studies on the test species were not available for OP.

Differences between the relative potencies of each of the compounds tested in this study are also described in the literature. These data are reviewed in Table 3, which reflects the differences in the potency of each of the chemicals tested. The potency of each chemical relative to E_2_ also varied extensively between studies. The cause of this variability is unknown, but is likely to reflect differences between the exposure systems, the concentrations tested, and the effect levels used to determine potency. Differences in species sensitivity may have also influenced the patterns observed.

### Mixture effects.

The results of the first mixture experiment demonstrate that mixtures of estrogenic chemicals have the capacity to act in combination and that their effects can be accurately predicted on the basis of the concentration–response curves of the individual mixture components according to the principles of CA. The predictions were in close agreement with the observed effects across the entire range of effects. Thus, we can conclude that the combined effect of the mixture does not deviate from additivity. This is consistent with the *a priori* assumption of this concept, which is dependent upon the components of the mixture acting via a common mechanism to contribute to the overall mixture effect. Although the validity of this concept has been demonstrated for estrogenic chemicals in assays involving unicellular organisms and mammalian cells (Payne et al. 2000, 2001; Rajapakse et al. 2002; Silva et al. 2002), these results provide the first evidence that the principles of CA hold true for multicomponent mixtures of estrogenic chemicals at higher organizational levels, despite the increased biological complexity of the assay system and the greater potential for toxicokinetic effects.

Similar additive effects have previously been reported in response to binary mixtures of estrogenic chemicals *in vivo*. Thorpe et al. (2001) investigated the effects of two-component mixtures on VTG induction in rainbow trout. Concentration–response curves were determined for fixed-ratio binary mixtures of E_2_ and NP (1:1,000) and of E_2_ and methoxychlor (MXC; 1:1,000), and these were related to the predictions of CA. The mixture of E_2_ and NP induced effects that were in agreement with the predictions of CA across the entire range of concentrations tested. In contrast, the mixture of E_2_ and MXC induced effects that were less than additive. This was attributed to the fact that MXC may act via a mechanism different from that of E_2_ and NP. Nevertheless, the effects observed provide strong evidence of the capacity for mixtures of similarly acting chemicals to behave in an additive manner according to the principles of CA. However, this conclusion was not confirmed in a subsequent investigation into the combined effects of E_2_ and EE_2_ (Thorpe et al. 2003). The effects of this mixture were consistent with CA at low-effect concentrations, but a divergence occurred with increasing effect level, with the predicted effects exceeding those that were observed. This was attributed to the limitations of the experimental design rather than being the result of a real deviation from additivity (Thorpe et al. 2003).

The problems encountered by Thorpe et al. (2003) were attributed to the fact that only three concentrations of the mixture were tested. This may have reduced the accuracy of the concentration–response relationship. An additional problem arose because of difficulties in defining the maximum response to the individual test compounds, as well as the maximum response predicted by CA. These difficulties were overcome in the present study by testing a wider range of mixture concentrations and by standardizing the response across exposures according to the minimum and maximum response of the controls. The accuracy with which these methods allowed the effects of the mixture to be predicted undoubtedly reflects the power of the mathematical modeling and statistical analyses. It also demonstrates the capacity for the VTG induction assay to produce high-quality, reproducible data for analyzing the mixture response.

### Low-dose implications.

The additive nature of the combined effects observed in the first mixture experiment demonstrates that all components contribute to the overall effect of a mixture. This implies that the overall effects will always exceed the highest individual effect of the mixture components. By this line of reasoning, low-effect concentrations of the individual components may give rise to considerable mixture effects. This phenomenon is of particular importance for the environmental hazard assessment of chemicals because it indicates that concentrations of chemicals that show no effect when applied singly may provoke substantial effects when acting in combination. The second mixture experiment investigated whether these theoretical conclusions from the CA concept also hold true in the real world, by analyzing the combined effect of the mixture components when they were present at low, noneffective concentrations. Even under these circumstances, a highly significant mixture effect of more than 50% was observed. These *in vivo* results were consistent with the “something from nothing” effects reported by Silva et al. (2002), which were produced using *in vitro* techniques.

More recently, the potential for estrogenic mixture effects at low concentrations has been explored *in vivo* using an assay based on an increase in rat uterotrophic weight (Tinwell and Ashby 2004). Concentrations that individually induced low effects were determined for seven estrogenic chemicals. Equipotent concentrations were tested, both individually and in combination, at various concentrations. The highest concentration of the mixture induced a significant increase in uterine weight in relation to the effects produced by the individual chemicals (although this difference was marginal). At 5- and 10-fold dilutions, few of the individual chemicals induced a significant response, and at a 50-fold dilution, no significant responses were observed. However, the same dilutions of the mixture were found to induce a significant response, thereby demonstrating the potential for mixture effects, even when the effects of each individual chemical cannot be detected. Although these findings were not related to expectations based on additivity, they are in perfect agreement with the results of the present study. This provides strong evidence of the capacity for estrogenic chemicals to act in combination at higher levels of biological organization, even at the type of low-effect concentrations encountered in the environment.

### Regulatory context.

Our findings in this study, combined with those of Tinwell and Ashby (2004), highlight the limitations of existing approaches to environmental (and human) risk assessment when considering the hazard posed by mixtures of endocrine-disrupting chemicals. Estrogenic chemicals, such as the alkylphenols, which are generally present in the environment as mixtures and at concentrations below those required to individually induce an effect, may therefore add to the overall risk when present with other chemicals that act via a similar mechanism. The failure to account for the combined effects of these chemicals will undoubtedly lead to the underestimation of potential hazards and hence erroneous conclusions regarding the risk that they pose. In demonstrating the inadequacy of the chemical-by-chemical approach to risk assessment, these findings represent a significant step toward achieving a more realistic means of assessing the environmental hazard posed by estrogenic chemicals. In addition to their regulatory implications, these findings indicate that CA may be a valuable tool for predicting the hazard posed by this type of mixture.

### Research needs.

It is important to recognize that CA can be applied only when the mixture is completely defined in terms of the number of chemicals present and the mixture ratio. A predictive risk assessment of combination effects will therefore depend heavily on the generation of robust tools for analyzing the type of mixtures that occur in real exposure situations. It should also be acknowledged that the scope of these findings is limited to the assessment of chemicals that act via the same mechanism to induce a common effect. The next major challenge will be to consider the endocrine-disrupting effects of mixtures of chemicals that act via different modes of action, or that have both agonistic and antagonistic effects. Potential interactions with non-endocrine-active compounds, such as solvents and surfactants, should also be considered, along with the influence of additional stresses incurred via changes in the environment and organismal physiology. Although the task of integrating this body of knowledge into hazard assessment procedures presents a formidable challenge, these improvements will be essential in ensuring the adequate protection of wildlife populations and human health.

## Figures and Tables

**Table 1:** Nominal and measured exposure concentrations at the beginning of each mixture experiment.

	EE_2_ (ng/L)		E_2_ (ng/L)		NP (μg/L)		OP (μg/L)		BPA (μg/L)	
Concentration (mixture dilution)	Nominal	Measured	Nominal	Measured	Nominal	Measured	Nominal	Measured	Nominal	Measured
First mixture experiment										
10.1 mg/L (5%)	0.03	0.03, 0.05	1.25	< 0.8, 1.3	0.35	0.4, 0.7	2.25	1.5, 2.4	7.5	4.1, 6.1
20.2 mg/L (10%)	0.06	0.07, 0.08	2.5	< 1.5, 2.6	0.7	0.7, 0.8	4.5	2.5, 5.1	15	9.6, 12
40.4 mg/L (20%)	0.12	0.14, 0.19	5	3.9, 4.9	1.4	0.9, 1.4	9	4.5, 8.2	30	19, 22
60.6 mg/L (30%)	0.18	0.23, 0.23	7.5	6.2, 9.0	2.1	2.3, 2.0	13.5	11, 12	45	43, 32
101 mg/L (50%)	0.3	0.31, 0.42	12.5	13, 16	3.5	3.5, 2.8	22.5	20, 14	75	79, 41
202 mg/L (100%)	0.6	0.6, 1.0	25	25, 28	7	7.1, 5.5	45	35, 32	150	150, 110
Second mixture experiment										
40.4 mg/L (20%)	0.12	0.13	5	6	1.4	1.8	9	9.4	30	20

The measured values given for the first mixture experiment represent the concentrations determined during two independent exposure studies.

**Table 2:** VTG induction by the individual compounds and the mixture.

	Concentration–response function				
Compound	Model*^a^*	β̂_1_	β̂_2_	σ̂^2^between exp	EC_50_ (95% CI)
EE_2_	Probit	5.03	1.65	0.29	0.0009 (0.0005–0.001)
E_2_	Probit	3.75	2.33	0.11	0.025 (0.020–0.029)
NP	Logit	−7.10	8.40	< 10^6^	7.02 (6.05–8.56)
OP	Weibull	−6.37	3.57	< 10^6^	48.2 (36.2–58.0)
BPA	Probit	−5.61	2.55	0.06	158 (119–205)
Mixture					
Observed	Weibull	−6.61	3.71	< 10^6^	48.0 (40.9–61.4)
Predicted	CA	—	—	—	44.3 (38.6–47.1)

CI, confidence interval. β̂_1_ and β̂_2_ are statistical estimates of model parameters; 95% CIs are approximate confidence intervals for effect concentrations given in μg/L; σ̂_2_ between exp is the statistical estimate for variance between experiments; and EC_50_ values are in relation to the NCs and PC, calculated from the given concentration–response function (rounded values).

a Concentration–response functions as defined by Scholze et al. (2001).

**Table 3:** Relative potencies previously reported for the five mixture components in terms of VTG induction.

Test organism	Sex	Exposure system	Exposure duration (days)	Effect level	EE_2_	E_2_	NP	OP	BPA
Roach (*Rutilus rutilus*)*^a^*	Male	Flow-through	21	LOEC	—	1	—	1,000	—
Rainbow trout (*Oncorhynchus mykiss*)*^a^*	Male	Flow-through	21	LOEC	—	1	—	100	—
Zebrafish (*Danio rerio*)*^b^*	Male	Flow-through	8	LOEC	0.06	1	—	—	—
Sheepshead minnow (*Cyprinodon variegatus*)*^c^*	Male	Flow-through	16	LOEC	0.53	1	50	—	—
Killifish (*Fundulus heteroclitis*)*^d^*	Male	Injection	8	LOEC	—	1	20	200	100
Rainbow trout (*Oncorhynchus mykiss*)*^e^*	Female (juvenile)	Flow-through	14	EC_50_	0.04–0.09	1	1,000	—	—
Zebrafish (*Danio rerio*)*^f^*	Male	Semistatic	21	LOEC	> 0.25	1	25,000	5,000	50,000
Rainbow trout (*Oncorhynchus mykiss*)*^f^*	Juvenile	Semistatic	21	LOEC	> 0.25	1	5,000	1,500	50,000
Fathead minnow (*Pimephales promelas*)*^g^*	Male	Flow-through	14	EC_50_	0.036	1	280	1,800	6,000

LOEC, lowest observed effect concentration. These data are scaled relative to the E_2_ potency observed in each study.

a Routledge et al. (1998).

b Rose et al. (2002).

c Folmar et al. (2003).

d Pait and Nelson (2003).

e Thorpe et al. (2001).

f Van den Belt et al. (2003).

g Present study.
